# Radiotherapy for nonagenarians: the value of biological versus chronological age

**DOI:** 10.1186/s13014-020-01563-x

**Published:** 2020-05-19

**Authors:** Tanja Sprave, Alexander Rühle, Raluca Stoian, Alina Weber, Constantinos Zamboglou, Carsten Nieder, Anca-Ligia Grosu, Nils H. Nicolay

**Affiliations:** 1 Department of Radiation Oncology, University of Freiburg - Medical Center, Robert-Koch-Str. 3, 79106 Freiburg, Germany; 2 German Cancer Consortium (DKTK) Partner Site Freiburg, German Cancer Research Center (dkfz), Neuenheimer Feld 280, 69120 Heidelberg, Germany; 3 Department of Oncology and Palliative Medicine, Nordland Hospital, 8092 Bodø, Norway; 4 Department of Clinical Medicine, Faculty of Health Sciences, UiT - The Arctic University of Norway, Tromsø, Norway

**Keywords:** Radiotherapy, Chemotherapy, Elderly patients, Geriatric patients, Comorbidities

## Abstract

**Background:**

The number of nonagenarian cancer patients (≥ 90 years) is continuously increasing, and radiotherapy is performed in a relevant proportion of patients, as surgery and chemotherapy are often not feasible for these patients. However, the evidence regarding the feasibility and treatment outcomes after radiotherapy for this patient group is very limited.

**Methods:**

All nonagenarian patients receiving (chemo) radiotherapy between 2009 and 2019 at the University of Freiburg - Medical Center were analyzed for patterns of care, overall survival (OS) and therapy-associated toxicities according to the Common Terminology Criteria for Adverse Events. Uni- and multivariate Cox regression analyses were conducted to assess the influence of patient- and treatment-related factors on patient outcomes.

**Results:**

One hundred nineteen patients with a total of 137 irradiated lesions were included in this analysis. After a median follow-up of 27 months, median OS was 10 months with a 3-year OS amounting to 11.1%. Univariate analyses demonstrated that a reduced performance status (HR = 1.56, 95% CI 1.00–2.45, *p* < 0.05), a higher burden of comorbidities (HR = 2.00, 95% CI 1.00–4.10, *p* < 0.05) and higher UICC tumor stages (HR = 2.21, 95% CI 1.14–4.26, *p* < 0.05) were associated with impaired survival rates. Split-course treatments (HR = 2.05, 95% CI 1.07–3.94, *p* < 0.05), non-completion of radiotherapy (HR = 7.17, 95% CI 3.88–13.26, *p* < 0.001) and palliative treatments (HR = 2.84, 95% CI 1.68–4.81, *p* < 0.05) were found to result in significantly reduced OS. In the multivariate analysis, split-course concepts (HR = 2.21, 95% CI 1.10–4.37, *p* < 0.05) and palliative treatments (HR = 3.19, 95% CI 1.77–5.75, *p* < 0.001) significantly deteriorated outcomes, while impaired ECOG status (HR = 1.49, 95% CI 0.91–2.43, *p* = 0.11) did not. The vast majority of patients reported either no (*n* = 40; 33.6%) or grade 1–2 acute toxicities (*n* = 66; 55.5%), and only very few higher-grade toxicities were observed in our study.

**Conclusion:**

Radiotherapy for nonagenarian patients is generally feasible and associated with a low toxicity profile. Given the relatively poor OS rates and the importance of the quality of life for this patient group, individualized treatment regimens including hypofractionation concepts should be considered.

## Introduction

The so-called “oldest-old” population, which includes people exceeding 85 years according to the United States National Institute of Aging, is continuously rising globally [1]. While there were about 100,000 centenarians (≥ 100 years) in 1990, this number quadrupled to more than 450,000 in 2015 and is estimated to more than 25 million in 2100 [2]. In line with the rising life expectancy, the number of elderly cancer patients is continuously increasing in Western countries; however, incidence and cancer mortality has been shown to decrease at the oldest ages [3]. As elderly and especially very old patients are commonly excluded in clinical trials, treatment decisions for this patient cohort are challenging. Due to the high incidence of comorbidities, polypharmacy and functional incapacities, nonagenarian cancer patients (≥ 90 years) comprise an especially vulnerable population. Several studies have shown that the probability of elderly cancer patients to receive state-of-the-art treatments is significantly lower than for younger patients even with equal cancer stage and comorbidities [4, 5]. Despite a high prevalence of frailty and comorbidities, there is a distinct subgroup of relatively fit nonagenarians, for whom curative treatments may be reasonable. As a patient’s chronologic age alone provides insufficient information regarding the optimal treatment decision, geriatric assessments have proven their value in predicting treatment tolerance and compliance [6]. Whereas more and more studies have demonstrated both the feasibility and the effectiveness regarding radiotherapy for elderly patients, radiotherapy for patients aged ≥90 years is poorly studied in the literature [7–10]. Although there is a limited number of retrospective studies analyzing the treatment outcomes of radiotherapy for nonagenarian patients, evidence regarding radiotherapy for this patient group remains poor due to relatively low patient numbers in most of these studies [11–20].

Here, we present the outcome and toxicity data from one of the largest analysis regarding radiotherapy for nonagenarians. In this retrospective study, we analyzed demographic parameters, radiotherapy characteristics, oncological outcomes and toxicity results in 119 patients aged 90 years and older who received (chemo) radiotherapy for various malignancies between 2009 and 2019 in a tertiary cancer center. Additionally, risk factors correlating with impaired survival rates were assessed in these patients.

## Material and methods

### Patients and treatment

All cancer patients aged ≥90 years who received radiotherapy between 2009 and 2019 at the University of Freiburg Medical Center were included in this analysis. Demographic characteristics, radiotherapy specifications and clinical data including toxicity reports were retrospectively surveyed using the electronic patient records. Comorbidities were quantified using the validated Adult Comorbidity Evaluation 27 (ACE-27) index, as it has been shown that comorbidity is an independent prognostic factor for cancer patients [21]. Tumor classification of most solid tumors was encoded according to the 8th edition of the UICC TNM classification, while brain tumors were encoded according to the WHO Classification of Tumors of the Central Nervous System. The Ann-Arbor classification was used for non-Hodgkin lymphoma, and myeloma was classified in accordance with the Salmon-Durie classification. The Independent Ethics Committee of the Medical Faculty, University of Freiburg approved this analysis (record no. 376/19).

### Radiation treatment

**Fig. 1:**
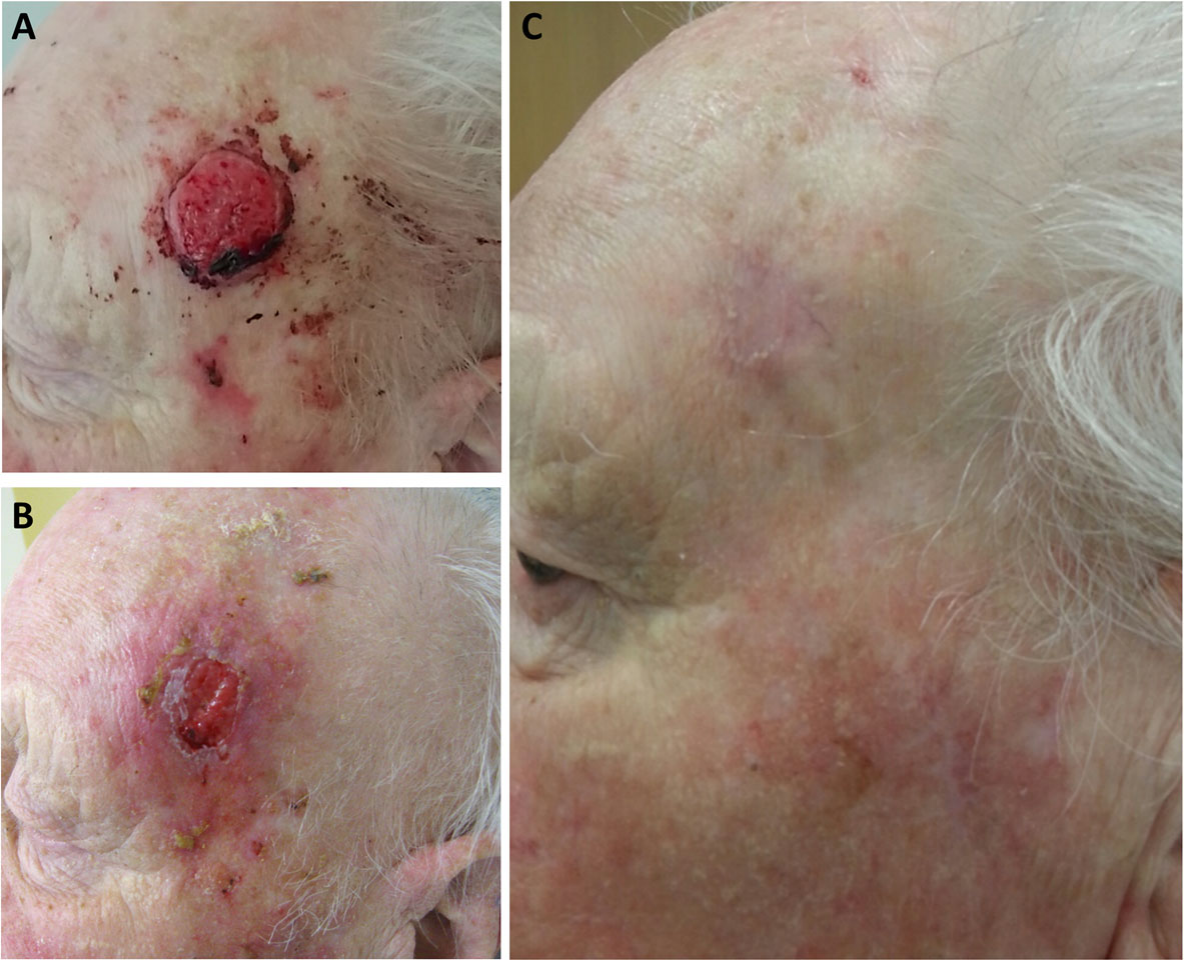
Clinical case of a 94-year old male patients who received radiotherapy for a well-differentiated squamous cell carcinoma. **a** Pre-therapeutic situation in November 2015 showing an ulcerating and bleeding skin lesion on the left temple. Radiotherapy was performed using electron beams with a total of 51 Gy (27 Gy applied in 9 fractions followed by 24 Gy in 6 fractions). **b** Irradiation-induced dermatitis (grade 2) in December 2015 after completion of treatment. **c** Follow-up consultation in April 2016 showing a complete clinical response with no high-grade radiotherapy-related toxicities

Depending on the time period of treatment and tumor localization, patients were treated with intensity-modulated radiotherapy (IMRT), three-dimensional conformal radiotherapy (3D radiotherapy) or electron beam radiotherapy for superficial skin cancer lesions (Fig. 1). Oncentra MasterPlan® (Nucletron BV, Veenendaal, the Netherlands) and Eclipse™ planning systems (Varian Medical Systems) were used for radiotherapy planning. Depending on the target volume and primary malignancy, individual concepts regarding the gross tumor volume (GTV), clinical target volume (CTV) and planning target volume (PTV) were applied according to the institutional guidelines. Treatments using moderate hypofractionation were usually applied for adjuvant breast cancer radiotherapy (e.g. 40.05 Gy applied in 15 fractions), painful bone metastases (30 Gy or 36 Gy in 10 fractions or 8 Gy in 1 fraction) or highly palliative situations such as bleeding gynaecological tumors (30 Gy in 10 fractions or 20 Gy in 5 fractions) in our cohort. Normofractionated treatments were typically used for definitive treatments with curative intention, e.g. for non-melanoma skin cancers (60 Gy in 30 fractions). Few patients received stereotactic body radiotherapy (SBRT) for primary or secondary lung tumors; in these cases, 35 Gy in 5 fractions or 37.5 Gy in 3 fractions were used. Radiotherapy characteristics are summarized in Table 1.

**Table 1:** Treatment details regarding radiotherapy and concomitant systemic treatment (*n* = 119 patients with 137 lesions)

		n	%
**Radiotherapy completion**	completed	99	83.2
	discontinued	20	16.8
**Split course treatment**	no split-course	123	89.8
	split-course	14	10.2
**Simultaneous integrated boost (SIB)**	no SIB	130	94.9
	SIB	7	5.1
**Radiotherapy technique**	3D	94	68.6
	IMRT/VMAT	26	19.0
	3D + IMRT	6	4.4
	Electrons	11	8.0
**Radiotherapy intention**	curative	38	31.9
	palliative	81	68.1
**Location**	cranial	11	8.0
	head-and-neck	49	35.8
	thoracic	21	15.3
	abdomen	4	2.9
	pelvis	28	20.4
	extremity	6	4.4
	spinal column	18	13.1
**Chemotherapy**	no chemotherapy	110	92.4
	chemotherapy	9	7.6
**Endocrine therapy**	no endocrine therapy	92	77.3
	endocrine therapy	27	22.7
		**median**	**range**
**Radiotherapy specification**	number of fractions	14	1–33
	single fraction dose	2.5	1.7–18.75
	total dose	39.0	1.8–72.0

### Survival and acute toxicities

For curative radiotherapy, follow-up consultations were performed regularly, whereby the intervals varied depending on the primary malignancy and patient wishes. Patients who were treated in a palliative intention commonly had at least one follow-up consultation at 6 weeks after completion of radiotherapy in order to evaluate the treatment response and acute toxicities. OS was defined as the time span from the start of radiotherapy to the last follow-up or death, whereby censoring was applied for patients who were lost to follow-up. Missing survival data were obtained by contacting the state authorities of Baden-Württemberg, Germany. Acute radiotherapy-related toxicities were retrospectively classified based on the Common Terminology Criteria for Adverse Events (CTCAE) v.5.0.

### Statistical analyses

The Kaplan-Meier method was applied to determine the OS of the study population, and the survival curves stratified by different clinical factors were compared using log-rank tests. The median follow-up in the study cohort was calculated using the reverse Kaplan-Meier method. Both univariate and multivariate Cox regression analyses were conducted in order to evaluate the influence of patient-related and clinical parameters on the survival. Pre-therapeutic parameters which proved to be significant in the univariate analysis (ECOG status, ACE-27 score, split-course concept, treatment intention (palliative vs. curative), disease stage and hospitalization status) were included in the multivariate analysis, in which a backward stepwise Cox regression with likelihood ratio (LR) tests was used. Chi-square tests were used to test for associations between categorical variables. Statistical significance was assumed for *p*-values < 0.05. IBM SPSS Statistics software version 22 (IBM, Armonk, NY, USA) was used for all statistical analyses.

## Results

### Patient and treatment characteristics

One hundred nineteen nonagenarian patients underwent radiotherapy between 2009 and 2019 and were included in our analysis. In line with an increased female-to-male-ratio in this elderly age group, 81 female patients (68.1%) and 39 male patients (31.9%) received radiotherapy (Table 2). The age in the study cohort ranged between 90 and 99 years with a median age of 91 years. The majority of patients demonstrated a reasonably good performance status with ECOG 1 (*n* = 58, 48.7%) being most prevalent. However, about one third of the study cohort was found to have a performance status ranging between ECOG 2 and ECOG 3. Existing comorbidities prior to treatment initiation were quantified according to the ACE-27 index and revealed that the large majority of the study population exhibited moderate (ACE-27-score of 2; *n* = 28, 23.5%) or severe (ACE-27-score of 3; *n* = 53, 44.5%) comorbidities. Skin cancers including basal cell carcinoma, squamous cell carcinoma and melanoma were the most common primary tumors in our study with 44 patients (37.0%) suffering from these malignancies, followed by breast cancer (*n* = 15, 12.6%) and other gynecological malignancies (*n* = 11, 9.2%) (detailed diagnoses are listed in Table 2). The majority of patients (*n* = 61, 51.3%) exhibited UICC stage IV tumors, followed by stage III (*n* = 20, 16.8%), stage II (*n* = 13, 10.9%) and stage I tumors (*n* = 10, 8.4%). Whereas 38 (31.9%) patients received curative radiotherapy, 81 (68.1%) were treated in a palliative setting. Fifty-four patients (45.4%) were treated on an outpatient basis, and 65 patients (54.6%) received their treatment as inpatients. On average, inpatients were hospitalized for a median of 16 days (range 2–43 days), and the majority of inpatients were discharged home (*n* = 49, 75.4%) or to nursing homes (*n* = 11, 16.9%) after radiotherapy.

**Table 2:** Patient characteristics of 119 nonagenarian patients receiving radiotherapy between 2009 and 2019

		n	%
**Sex**	male	38	31.9
	female	81	68.1
**Age**	90–94 years	101	84.9
	95–99 years	18	15.1
**ECOG**	0	15	12.6
	1	58	48.7
	2	36	30.3
	3	7	5.9
	missing	3	2.5
**ACE-27**	0	12	10.1
	1	26	21.8
	2	28	23.5
	3	53	44.5
**Malignancy**	skin	44	37.0
	breast	15	12.6
	other gynecological^1^	11	9.2
	prostate	8	6.7
	lymphoma	7	5.9
	head-and-neck	6	5.0
	urological cancer^2^	6	5.0
	lung	4	3.4
	myeloma	3	2.5
	anal	3	2.5
	others^3^	4	3.4
**Tumor stage**	UICC I	10	8.4
	UICC II	13	10.9
	UICC III	20	16.8
	UICC IV	61	51.3
	Ann-Arbor I	1	0.8
	Ann-Arbor II	2	1.7
	Ann-Arbor IV	4	3.4
	Salmon-Durie I	2	1.7
	Salmon-Durie II	1	0.8
	WHO-grade (brain tumor)	1	0.8
	unknown	4	3.4
**Inpatient stay**	inpatient	65	54.6
	outpatient	54	45.4
**Discharge**	home	49	75.4
	nursing home	11	16.9
	others	5	7.7

^1^vulva, cervix, uterus ^2^kidney, ureter, bladder ^3^gall bladder, sarcoma*,* meningeoma

Ninety-nine patients (83.2%) completed their prescribed course of radiotherapy, while 20 patients (16.8%) discontinued their treatment, most commonly due to a deterioration of their general condition. A split-course radiotherapy concept was applied in 14 patients (10.2%), and 3 patients (21.4%) of these did not receive the intended radiotherapy dose. Most patients were treated in the head-and-neck region (*n* = 49, 35.8%), followed by the pelvic (*n* = 28, 20.4%) and thoracic regions (*n* = 21, 15.3%). About one in four patients (*n* = 27, 22.7%) was treated with hormonal therapy for either breast or prostate cancer, while only 9 patients (7.6%) received concomitant chemotherapy. Treatment was administered to a median dose of 39.0 Gy (range 1.8 Gy – 72.0 Gy), and patients received a median of 14 fractions (range 1–33).

### Treatment outcomes

**Fig. 2:**
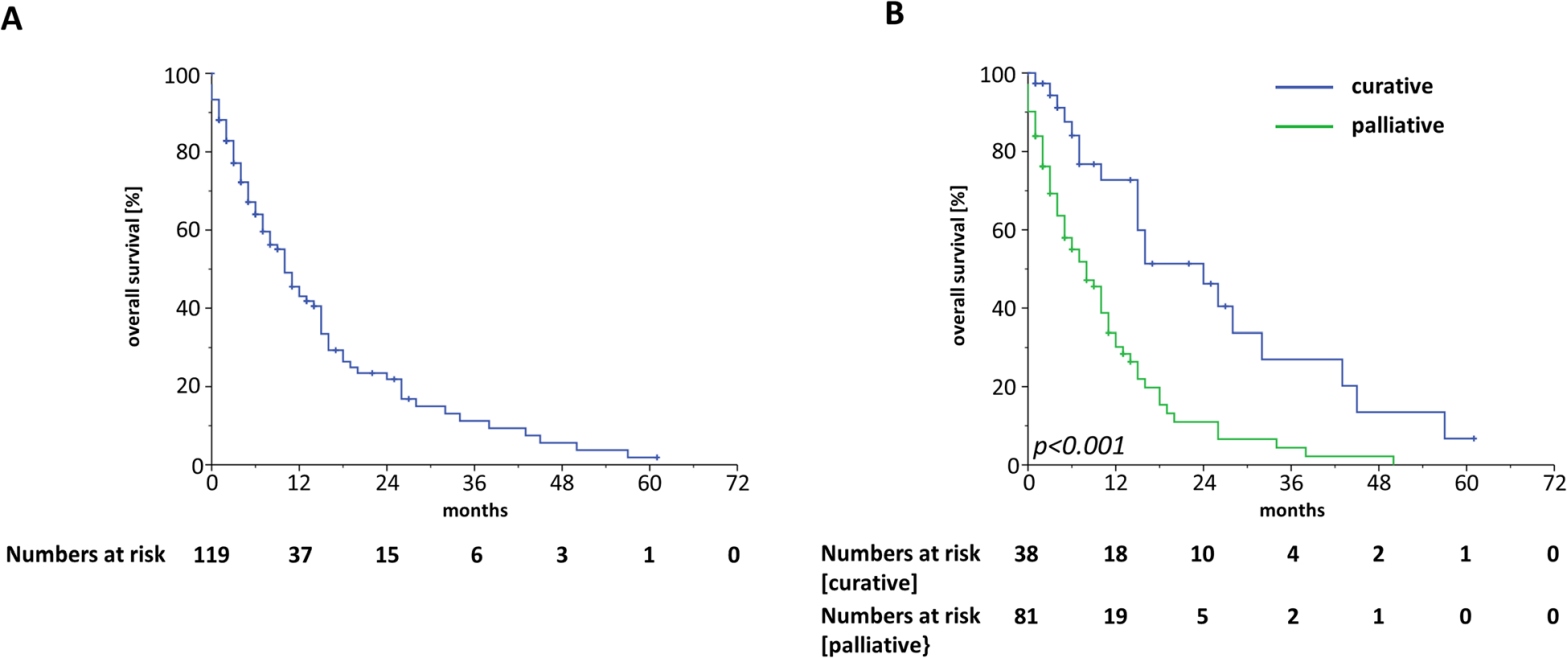
**a** Kaplan-Meier curves for OS of nonagenarian patients treated by radiotherapy (*n* = 119). **b** OS curves stratified by curative and palliative radiotherapy. Log-rank-tests were performed for statistical comparisons

**Fig. 3:**
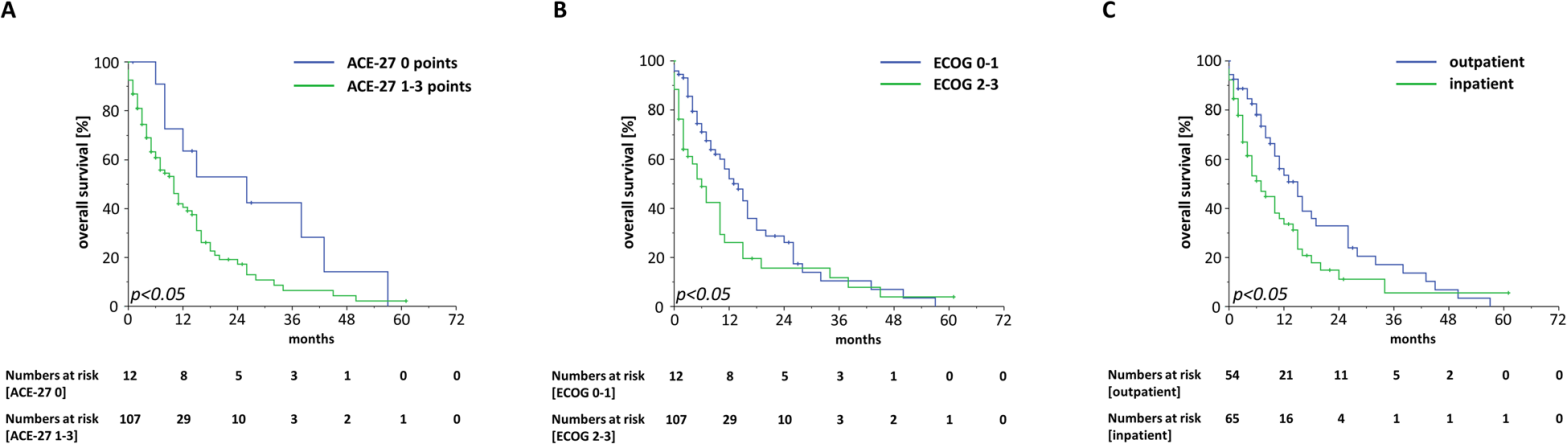
Kaplan-Meier OS curves regarding patients’ comorbidity burden (**a**), performance status (**b**), and hospitalization during radiotherapy (**c**)

**Fig. 4:**
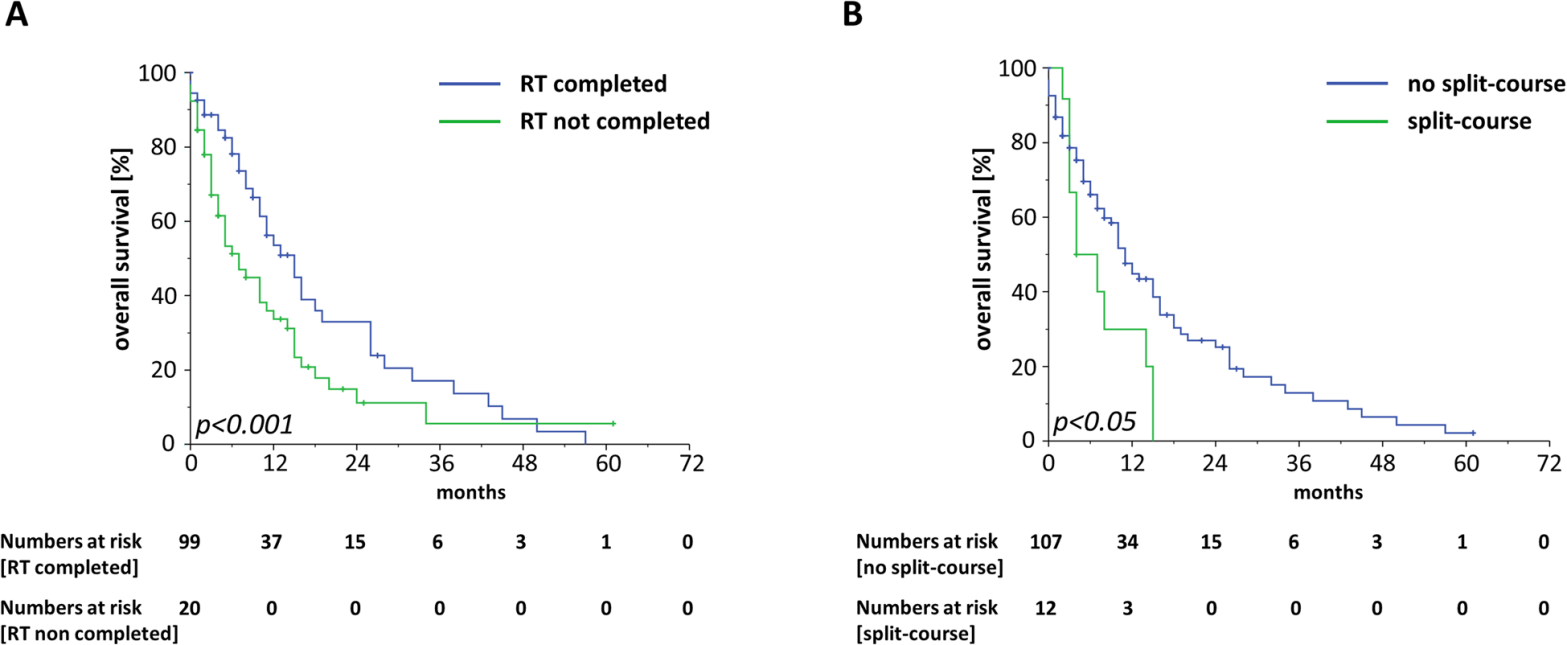
Kaplan-Meier curves for OS stratified by the radiotherapy completion status (**a**) and application of a split-course concept (**b**)

Median OS ranged at 10 months with a 1-year OS, 2-year OS and 3-year OS of 42.6, 21.6 and 11.1%, respectively (Fig. 2a). At 3 months after the start of radiotherapy, 76.8% of patients were still alive. Patients who were treated in a curative setting were shown to have superior OS rates compared to patients receiving palliative radiotherapy treatment (HR = 2.84, 95% CI 1.68–4.81, *p* < 0.001) (Fig. 2b, Table 3). The median OS for patients treated with curative intention was shown to be 24 months which was three times higher than the median OS of 8 months for patients receiving radiotherapy with palliative intention. Whereas survival was comparable between patients aged 90–94 years and patients ≥95 years (HR = 1.44, 95% CI 0.84–2.47, *p* = 0.19), higher values in the ACE-27 instrument as an indicator for a higher burden of comorbidities as well as a reduced performance status were found to be significantly associated with an impaired OS (ACE-score: HR = 2.00, 95% CI 1.00–4.10, *p* < 0.05; ECOG-status: HR = 1.56, 95% CI 1.00–2.45, *p* < 0.05) (Fig. 3a-b). Additionally, patients who were hospitalized during radiotherapy exhibited a lower OS than patients treated on an outpatient basis (HR = 1.64, 95% CI 1.05–2.66, *p* < 0.05) (Fig. 3c). Neither concomitant chemotherapy (HR = 1.02, 95% CI 0.32–3.26, *p* = 0.98) nor endocrine treatment (HR = 1.26, 95% CI 0.75–2.47, *p* = 0.19) influenced the survival rates after radiotherapy. Interestingly, skin cancer patients did not exhibit superior survival rates compared to non-skin cancer patients (HR = 0.87 in favor of non-skin cancer patients, 95% CI 0.56–1.36, *p* = 0.54) (Supplementary Figure 1A). Survival rates did significantly differ between the different UICC stages with the best OS rates for patients with UICC stage I (median OS of 43 months) and the worst OS rates for patients with UICC stage IV (median OS of 8 months) (UICC stage III-IV versus UICC stage I-II: HR = 2.21, 95% CI 1.14–4.26, *p* < 0.05) (Supplementary Figure 1B). Unplanned discontinuation (HR = 7.17, 95% CI 3.88–13.26, *p* < 0.001) due to worsening of a patient’s general condition as well as split-course concepts (HR = 2.05, 95% CI 1.07–3.94, *p* < 0.05) were observed to result in reduced survival rates (Fig. 4a-b).

**Table 3:** Univariate and multivariate analysis of clinical parameters regarding OS in nonagenarian patients receiving radiotherapy. Pre-therapeutic parameters which were significant in the univariate analysis were included in the multivariate analysis

	HR	CI 95%	***p***-value
**Univariate analysis**			
Age 95–99 years / 90–94 years	1.44	0.84–2.47	0.19
ECOG 2–3 / 0–1	1.56	1.00–2.45	0.05
ACE-27 1–3 / 0	2.02	1.00–4.10	0.05
Split-course / no split-course	2.05	1.07–3.94	0.03
Radiotherapy not completed / completed	7.17	3.88–13.26	0.00
Palliative treatment / curative treatment	2.84	1.68–4.81	0.00
No Chemotherapy / chemotherapy	1.02	0.32–3.26	0.98
No endocrine treatment / endocrine treatment	1.26	0.75–2.11	0.38
Non-skin cancer / skin cancer	0.87	0.56–1.36	0.54
UICC stage I – II / III – IV	2.21	1.14–4.26	0.02
Inpatient / outpatient	1.64	1.05–2.55	0.03
**Multivariate analysis**			
ECOG 2–3 / 0–1	1.49	0.91–2.43	0.11
ACE-27 1–3 / 0	2.37	0.91–6.18	0.08
Split-course / no split-course	2.21	1.10–4.37	0.02
Palliative treatment / curative treatment	3.19	1.77–5.75	0.00
UICC stage I – II / III – IV	1.68	0.76–3.74	0.20
Inpatient / outpatient	1.18	0.71–1.94	0.53

In the multivariate analysis of significant pre-therapeutic parameters, split-course treatments (HR = 2.21, 95% CI 1.10–4.37, *p* < 0.05) and palliative treatments (HR = 3.19, 95% CI 1.77–5.75, *p* < 0.001) remained significant risk factors for reduced OS. In contrast, the ECOG performance status (HR = 1.49, 95% CI 0.91–2.43, *p* = 0.11), the comorbidity burden (HR = 2.37, 95% CI 0.91–6.18, *p* = 0.08) and the hospitalization status (HR = 1.18, 95% CI 0.71–1.94, *p* = 0.53) were found to be not significant regarding OS upon multivariate analysis.

In order to analyze whether the comorbidity burden or ECOG performance status differed between the split-course- and continuous course-group, Chi-square tests were conducted and showed no imbalances regarding these parameters between both groups (ACE-score 0 versus 1–3: *p* = 0.43, χ2-test; ECOG-score 0–1 versus 2–3: *p* = 0.78, χ2-test).

### Toxicities

As the majority of patients received palliative treatments with reduced radiation doses, toxicity rates in our cohort were found to be relatively low. About one third of the study population (*n* = 40, 33.6%) did not report any radiotherapy-related acute toxicities, and 66 patients (55.5%) only exhibited CTCAE grade 1–2 adverse reactions (Table 4). Thirteen patients (10.9%) suffered from at least one grade 3 toxicity, most commonly dermatitis (*n* = 6), mucositis (*n* = 4) or pain (*n* = 3) (Supplementary Table 1). However, no grade 4–5 acute toxicities were observed in our study population. The median follow-up time and number of follow-up visits was insufficient to robustly assess chronic toxicities.

**Table 4:** Acute toxicity results after radiotherapy of 119 patients aged 90 years and older according to CTCAE

	n	%
CTCAE 0	40	33.6
CTCAE 1	47	39.5
CTCAE 2	19	16.0
CTCAE 3	13	10.9
CTCAE 4	0	0.0
CTCAE 5	0	0.0

## Discussion

In this analysis regarding the outcomes of radiotherapy for nonagenarian patients, we could demonstrate that radiotherapy was feasible and could be completed in about 85% of patients. The toxicity profile in our cohort was relatively low with only 13 patients (10.9%) suffering from grade 3 toxicities and no reported grade 4–5 toxicities.

However, the median OS of 10 months reflected the very advanced age and the resulting frailty of these patients. The median survival reported from our dataset is similar to previous studies regarding radiotherapy for nonagenarians which ranged between 9 and 23 months [11, 13]. The fact that two thirds of our population received palliative radiotherapy must be taken into consideration when comparing our outcomes with the results of a previous French multi-center study which reported a median OS of almost 2 years, but included considerably more patients undergoing curative treatments (44% versus 32% in our study) [13]. In a comparable analysis by Kocik et al., median OS of the entire nonagenarian cohort ranged at 9 months with palliative patients demonstrating an OS of only 3.6 months [11]. This significant survival differences between patients receiving curative and palliative radiotherapy is mirrored in our dataset: Patients who received radiation therapy with curative intention in our nonagenarian cohort exhibited a median OS of 24 months. It should be mentioned that 26 patients died within 3 months after the initiation of radiotherapy, whereby 16 of these patients had an ECOG performance status of 2–3 and 20 an ACE-27-score of 2–3. Of these 26 patients, moderate hypofractionation (single doses between 2 and 3 Gy) was applied in 15 cases and strong hypofractionation (single doses > 3 Gy) in 3 cases. Hypofractionation, especially in the palliative setting, should be strongly considered for all nonagenarian patients with a reduced performance status and a high burden of comorbidities [22, 23].

The significant impact of both the ECOG performance status and the ACE-27 comorbidity score on the survival rates in the univariate analysis underlines the value of cautious clinical assessments prior to radiotherapy initiation in very old patients. Assessment of the comorbidities using validated instruments such as the Charlson Comorbidiy index or the ACE-27 score are relatively easy to perform and may be used especially in hospitalized elderly patients. The prognostic value of comorbidities in elderly cancer patients has been shown for many tumor types such as lung cancer and head-and-neck cancers [24, 25]. In this context, standardized approaches that help to reduce intra- and interobserver variability have demonstrated an advantage compared to the assessment of a patient’s performance status by the treating physician [26]. Furthermore, it should be noted that only the ACE-27 comorbidity score but not the ECOG performance status remained a significant risk factor in the multivariate analysis of several pre-therapeutically available information. Based on our analysis, assessments of the patient’s comorbidities via validated instruments may be superior compared to the more subjective assessment of the patient’s performance status regarding survival prediction after radiotherapy.

Using the official mortality table provided by the Federal Statistical Office of Germany, the mean life expectancy of a general German cohort with the same age distribution as in our nonagenarian cancer cohort would amount to 42 months, which is more than 2 years longer than the mean OS of 15 months (corresponding to a median OS of 10 months) in our cohort [27]. These data demonstrate that even people older than 90 years exhibit an average remaining life expectancy of several years, showing that curation in very old cancer patients without life-threatening comorbidities may lead to some additional years of life.

In our study, concomitant chemotherapy was administered in 9 patients. In most cases, chemotherapy was initiated prior to radiotherapy and continued during the course of radiation treatment. Rivoirard and colleagues investigated the efficacy and safety of systemic treatments among nonagenarian cancer patients [28]. Although the general condition of these patients was relatively good and chemotherapy dosages were significantly reduced in about half of the patients, 8 of 12 patients experienced acute grade 3–4 toxicities, and 2 died of complications related to chemotherapy, thus demonstrating the difficulty of applying chemotherapy in this frail patient group. Besides the marked toxicity profile, oncological outcomes were relatively poor with overall survival ranging between 18 days and several years. In a retrospective analysis by Mitsuhashi et al., 32 patients aged above 90 years who received radiotherapy were analyzed [15]. Concomitant chemotherapy was administered only in patients with non-Hodgkin’s lymphoma, and the 2 patients who received simultaneous chemoradiotherapy died of chemotherapy-related pneumonitis within 1 month after radiotherapy. Therefore, chemotherapy, especially when applied concomitantly to radiotherapy, constitutes a major risk factor for treatment-related severe toxicities or death in these very old patients and should be indicated only with utmost caution.

Split-course treatments were used in about 10% of our nonagenarian patient cohort with the concept of decreasing toxicities; however, this concept was found to be associated with significantly impaired survival. It is commonly believed that split-course treatments could reduce radiotherapy-related toxicities, but may in turn result in impaired local control rates [29–32]. Delays in the course of radiotherapy have been shown to result in decreased locoregional control (LRC) rates for various malignancies such as head-and-neck cancer, bladder cancer and cervical cancer, likely due to accelerated repopulation effects [33–35]. Interestingly, 25% of patients with an intended split-course concept did not complete the planned radiotherapy course which was considerably higher than in our general cohort with a discontinuation rate of 16.8%. Potential confounder variables such as performance status or comorbidity burden did not differ between split-course and continuous-course-groups, and application of a split-course regimen remained a significant risk factor also in the multivariate analysis. However, the acute toxicity rate of the split-course cohort was found to be comparably low with only 1 patient developing a grade 3 mucositis and 2 patients suffering from a grade 2 dermatitis and mucositis, respectively.

SBRT was performed for 4 pulmonary lesions, whereby 3 lesions were primary lung cancers and 1 lesion a pulmonary metastasis. A query of the National Cancer Database identified 616 patients aged ≥90 with stage I non-small-cell lung cancer (NSCLC), and reported that about one third (202 patients) received local treatments with either surgery (75 patients) or SBRT (127 patients) [36]. Patients who underwent local therapies exhibited superior survival rates compared to patients who were treated using conventionally fractionated radiotherapy or patients receiving optimal supportive care. Interestingly, the survival of nonagenarian patients with stage-I NSCLC did not differ between the surgery and SBRT groups [36].

Owing to the fact that most patients received palliative radiotherapy with reduced irradiation doses as compared to definitive treatment concepts, only 13 patients (10.9%) developed any grade 3 acute toxicities. The high prevalence of patients treated on an inpatient basis (54.6%) may aid in preventing higher-graded toxicities, as early supportive care can help to ameliorate acute toxicities such as mucositis or dermatitis. With a median follow-up of only 27 months and a reduced availability of patients for regular follow-up examinations, chronic toxicities could not be sufficiently assessed in our nonagenarian cohort.

Despite providing survival and toxicity data obtained from one of the largest cohorts regarding radiotherapy for nonagenarian cancer patients, there are several limitations of our analysis: First, we were not able to calculate progression-free survival and locoregional control rates for patients treated with curative intention, as many patients were lost to follow-up soon after radiotherapy treatment, likely due to the very old age of these patients. Second, the impact of palliative radiotherapy on the patients’ quality of life or pain severity was not accessed in a sufficient number of patients, thereby impeding analyses about the effectiveness of palliative radiotherapy in our nonagenarian cancer cohort. Third, only patients that were deemed fit enough for treatment and therefore received radiotherapy were included in this analysis. This fact may help to explain the relatively good performance status in our cohort, and analyzed cohort may not be comparable to the general nonagenarian cancer population. Forth, the fact that we did not identify the patient’s age as a significant parameter in terms of OS may be related to the fact that about 70% of our patients were between 90 and 92 years old and only a minority of about 15% of patients exhibited an age of 95 years or older.

Nevertheless, radiotherapy is a feasible treatment modality for many nonagenarian cancer patients resulting in high treatment completion rates. The performance status and comorbidity burden were found to influence the survival rates after radiotherapy, showing the importance of routinely assessing these parameters as a basis for treatment decisions. Given the limited survival of patients with a low performance status and higher comorbidities, individualized, and shorter-course treatment regimens should be considered for these patients in order to reduce treatment and hospitalization times.

## Supplementary information

**Additional file 1: Supplementary Figure 1.** (**A**) Kaplan-Meier OS curves stratified by skin cancer and non-skin cancer patients. (**B**) Kaplan-Meier OS curves in dependence of the UICC stage.

**Additional file 2: Supplementary Table 1.** Acute toxicity results consisting several radiotherapy-related side reactions according to CTCAE. ^*1*^ e.g. *diarrhea, rectal bleeding*. ^*2*^ e.g. *dysuria, hematuria,* pollakiuri

## Data Availability

The datasets used and analyzed during the current study are available from the corresponding author on reasonable request.
