# Supplementary figures and images for: Radiotherapy for nonagenarians: the value of biological versus chronological age
Source: Radiat Oncol. 2020 May 19;15:113. doi: 10.1186/s13014-020-01563-x (PMC7236131; doi:10.1186/s13014-020-01563-x)

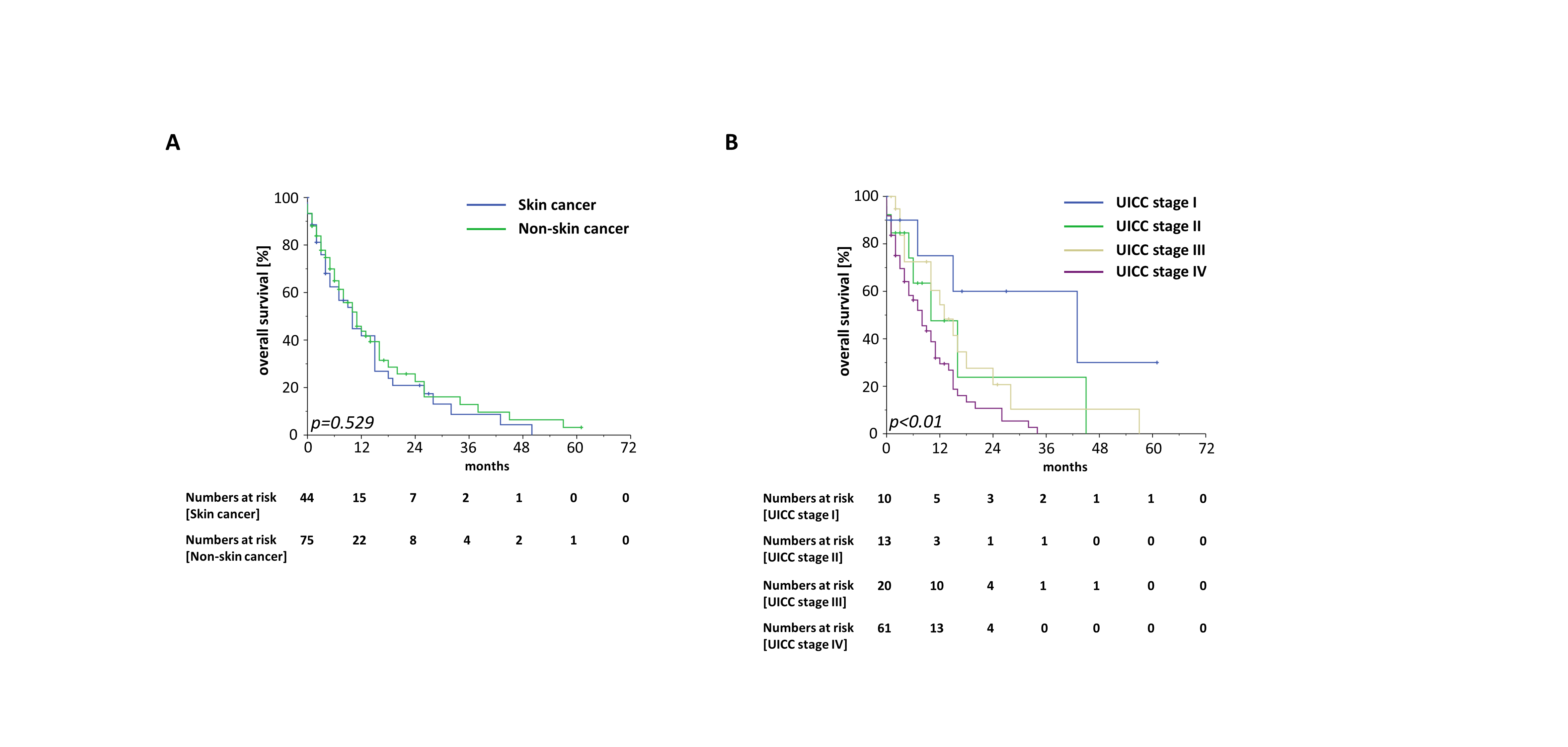

Supplement: Supplementary file 1 — Additional file 1: Supplementary Figure 1. (A) Kaplan-Meier OS curves stratified by skin cancer and non-skin cancer patients. (B) Kaplan-Meier OS curves in dependence of the UICC stage. [file 13014_2020_1563_MOESM1_ESM.tif]
