# Supplementary material for: Radiotherapy for nonagenarians: the value of biological versus chronological age
Source: Radiat Oncol. 2020 May 19;15:113. doi: 10.1186/s13014-020-01563-x (PMC7236131; doi:10.1186/s13014-020-01563-x)
Supplement: Supplementary file 2 — Additional file 2: Supplementary Table 1. Acute toxicity results consisting several radiotherapy-related side reactions according to CTCAE. 1 e.g. diarrhea, rectal bleeding. 2 e.g. dysuria, hematuria, pollakiuri [file 13014_2020_1563_MOESM2_ESM.docx]

Supplementary Table 1: Acute toxicity results consisting several radiotherapy-related side reactions according to CTCAE.

|  | **CTCAE grade** | | | | | |
| --- | --- | --- | --- | --- | --- | --- |
|  | **0** | **1** | **2** | **3** | **4** | **5** |
| dermatitis | 67 | 36 | 10 | 6 | 0 | 0 |
| dysgeusia | 111 | 8 | 0 | 0 | 0 | 0 |
| dysphagia | 102 | 14 | 3 | 0 | 0 | 0 |
| dyspnea | 117 | 1 | 1 | 0 | 0 | 0 |
| fatigue | 95 | 23 | 1 | 0 | 0 | 0 |
| gastrointestinal symptoms^1^ | 96 | 20 | 2 | 1 | 0 | 0 |
| genitourinary symptoms^2^ | 112 | 5 | 2 | 0 | 0 | 0 |
| mucositis | 100 | 7 | 8 | 4 | 0 | 0 |
| nausea | 114 | 5 | 0 | 0 | 0 | 0 |
| pain | 85 | 30 | 1 | 3 | 0 | 0 |

*^1^ e.g. diarrhea, rectal bleeding
^2^ e.g. dysuria, hematuria, pollakiuria*
